# Molecular Genetic Analysis of Orf Virus: A Poxvirus That Has Adapted to Skin

**DOI:** 10.3390/v7031505

**Published:** 2015-03-23

**Authors:** Stephen B. Fleming, Lyn M. Wise, Andrew A. Mercer

**Affiliations:** Department of Microbiology and Immunology, 720 Cumberland St, University of Otago, Dunedin 9016, New Zealand; E-Mails: lyn.wise@otago.ac.nz (L.M.W.); andy.mercer@otago.ac.nz (A.A.M.)

**Keywords:** parapoxvirus, orf virus, poxvirus evolution

## Abstract

Orf virus is the type species of the *Parapoxvirus* genus of the family *Poxviridae*. It induces acute pustular skin lesions in sheep and goats and is transmissible to humans. The genome is G+C rich, 138 kbp and encodes 132 genes. It shares many essential genes with vaccinia virus that are required for survival but encodes a number of unique factors that allow it to replicate in the highly specific immune environment of skin. Phylogenetic analysis suggests that both viral interleukin-10 and vascular endothelial growth factor genes have been “captured” from their host during the evolution of the parapoxviruses. Genes such as a chemokine binding protein and a protein that binds granulocyte-macrophage colony-stimulating factor and interleukin-2 appear to have evolved from a common poxvirus ancestral gene while three parapoxvirus nuclear factor (NF)-κB signalling pathway inhibitors have no homology to other known NF-κB inhibitors. A homologue of an anaphase-promoting complex subunit that is believed to manipulate the cell cycle and enhance viral DNA synthesis appears to be a specific adaptation for viral-replication in keratinocytes. The review focuses on the unique genes of orf virus, discusses their evolutionary origins and their role in allowing viral-replication in the skin epidermis.

## 1. Molecular Genetic Analysis of Orf Virus: A Poxvirus That Has Adapted To Skin

Skin is the largest organ of mammals and provides essential protection from injury and infection. The cellular immune system of skin and the associated lymphatic organs have developed from constant exposure to microbial pathogens during the course of evolution and as a consequence can respond rapidly and efficiently to such organisms [1,2]. Keratinocytes constitute approximately ninety percent of the cells within the epidermis and have evolved as immune sentinels of skin. The expression of a wide range of Toll-like receptors and other sensory molecules allows these cells to respond rapidly to infection by producing proinflammatory cytokines and interferons (IFNs) that are critical during the early innate immune responses and play a vital role in initiating the adaptive immune responses.

Orf virus (ORFV) is the type species of the *Parapoxvirus* genus and induces acute pustular skin lesions primarily in sheep and goats that are transmissible to man. ORFV infects keratinocytes and epithelial cells in the oral mucosa. In the last quarter of a century much progress has been made in understanding the interaction of this virus with its host. Molecular genetic studies have revealed that it has evolved a number of genes that are unique to the genus which have apparently allowed it to successfully infect these cells and to replicate in this highly tuned immune environment. A number of viral genes act within infected cells to manipulate the anti-viral host response. An anti-apoptotic factor, that has Bcl-2-like properties, prevents the induction of the cell death programme that the host employs to limit viral replication. A gene has been discovered that has a role in interferon resistance. Several of the genes limit inflammation either by suppressing the production of inflammatory factors from virus-infected cells or nearby cells. Three genes have been discovered that modulate the NF-κB signalling pathway. ORFV is known to encode several secreted soluble factors. An interleukin-10 (IL-10) like cytokine that suppresses the production of proinflammatory cytokines from activated cells and may have a role in impairing the development of the adaptive responses. A chemokine binding protein (CBP) that disrupts chemokine gradients thus blocking recruitment of immune cells to infected tissue from the dermis and blood and potentially inhibiting the movement of antigen presenting cells to nearby peripheral lymphoid organs where they initiate the adaptive immune responses. A granulocyte-macrophage colony-stimulating factor and interleukin-2 binding protein (GIF) is believed to have roles in blocking immune cell activation and growth. In addition ORFV encodes a vascular endothelial growth factor (VEGF) that induces blood vessel growth (angiogenesis) at the site of the lesion. The enhanced blood supply ensures a flow of nutrients and oxygen to cells at the skin surface. A further factor that has similarities to an anaphase promoting complex protein is believed to manipulate the cell cycle in infected cells so as to increase the nucleotide pool for viral DNA replication and to increase the abundance of metabolic enzymes for viral DNA synthesis. Phylogenetic analysis suggests that both the viral IL-10 and VEGF genes have been “captured” late from their host during the evolution of the parapoxviruses since they are not found in other poxvirus genera and show remarkable similarity to their cellular counterparts. It’s also likely that the anaphase promoting complex subunit-like gene and the anti-apoptotic factor gene have been captured from their host since these genes encode proteins that contain structural elements of mammalian proteins. Genes such as the CBP and GIF appear to have evolved from a common poxvirus ancestral gene while the NF-κB signalling pathway inhibitors have no homology to other known NF-κB inhibitors from other poxvirus genera. There still remain a number of genes in ORFV for which functions are not known and are unique to the *Parapoxvirus* genus. In this review we examine the genetic structure of ORFV and discuss its evolutionary relationships with other poxviruses. We examine the genes that are unique to this genus that allow it to manipulate metabolism and growth of keratinocytes and subvert the host’s defences so as to establish infection in the hostile environment of the skin.

## 2. Orf Virus

Orf virus (ORFV) is the prototype species of the *Parapoxvirus* genus of the *Poxviridae* family that includes *Pseudocowpox* (PCPV), *Bovine papular stomatitis virus* (BPSV), and the *Parapoxviruses of red deer in New Zealand* (PVNZ). Tentative members include *Sealpox virus*, *Ausdyk virus*, Parapoxvirus of reindeer and Chamois contagious ecthyma virus [3]. Several of the parapoxviruses are zoonotic pathogens including ORFV, BPSV and PCPV. Infection of humans by PVNZ has not been reported. All parapoxviruses induce acute cutaneous pustular lesions. The virions of parapoxviruses are characteristically ovoid. The criss-cross pattern seen by electron microscopy appears to be due to superimposed images of the tubule-like structure that is wound around the viral particle much like a ball of wool. This unique morphology has formed the basis for their inclusion as a separate group in the poxvirus family. Compared with other members of the poxvirus family, parapoxviruses have relatively small genomes with a high G+C content suggesting significant divergence from other genera of this family [4,5].

**Figure 1:**
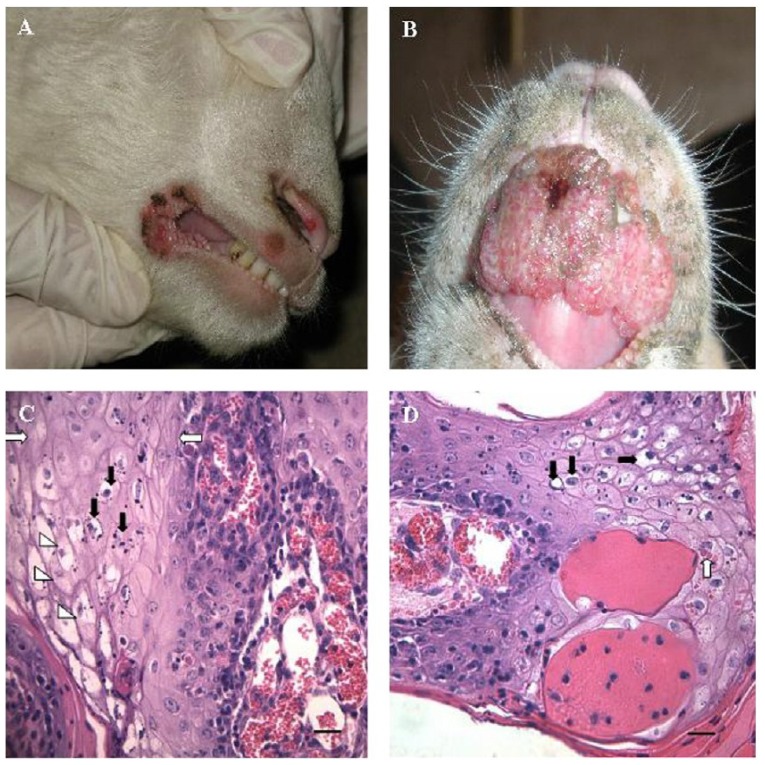
(**A**) Sheep showing multiple nodular lesions on the upper and lower labia and the junction of lips; (**B**) Sheep with severe proliferative orf lesions in the skin of lips and muzzle; (**C**) Spinous cells of the stratum spinosum showing acanthosis (white arrows), vacuolation (arrow heads) and karyorrhexis (black arrows). (H&E, 400×; bar = 100 µm); (**D**) Intraepithelial ballooning degeneration (black arrows) and intracytoplasmic eosinophilic inclusion bodies (white arrows) in the acanthocytes. (H&E, 400×; bar = 100 µm) (adapted from Zhao *et al.* [21] with permission from Elsevier publishing).

The natural hosts of ORFV are sheep and goats [6], however infections have occasionally been reported in camels [7], Japanese serow [8,9] and cats [10,11]. The virus is present in sheep and goat producing countries world-wide. In its natural hosts the disease caused by ORFV is commonly known as contagious pustular dermatitis, scabby mouth, sore mouth or orf [3]. In humans the lesions remain localised and infections on the hands are relatively common in people working in close contact with animals in the sheep industry. In immune impaired individuals, large highly vascularised tumour-like lesions of the skin have been reported [12,13]. ORFV usually infects the host through breaks and abrasions to the skin and replicates in regenerating keratinocytes [14]. There is no evidence of systemic spread of the virus [6]. ORFV lesions are normally benign, however, more serious complications can occur from secondary infections in their natural hosts by bacteria or fungi. In sheep and goats the disease is characterised by inflammatory proliferative pustular lesions affecting the skin, lips muzzle nostrils and oral mucosa (Figure 1A,B) [15]. Infection of the buccal cavity of sheep with ORFV results in a papulo-erosive stomatitis affecting the gums, palate and tongue [16]. ORFV lesions evolve through stages of macule, papule, vesicle, pustule scab and resolution [3]. The benign lesions resolve in approximately 6-8 weeks.

The histopathological features of the infected skin are characterised by vascularisation and the swelling of the keratinocytes in the stratum spinosum, reticular regeneration and marked epidermal proliferation (Figure 1C,D) [14,17,18,19]. Epidermal proliferation leads to markedly elongated rete pegs. Neutrophils migrate into areas of reticular regeneration giving rise to microabscesses that rupture on the surface. The histopathology of the underlying dermis includes oedema, marked capillary dilation and infiltration of inflammatory cells. Papillomatous growths often develop in natural ORFV infections [20].

## 3. Immune Response to Orf Virus

An intriguing phenomenon of ORFV is that animals can be reinfected with the virus either by experimental or natural infection although the size and severity of lesions diminishes with each occurrence. Furthermore the live virus vaccine propagated in sheep [17,22] and attenuated virus produced in cell culture [22,23,24] elicits only short-term protective immunity of approximately 6–8 months [6]. In view of the ability of the virus to reinfect its host and the deficiencies of the vaccine, several laboratories have undertaken studies to investigate the immune response against ORFV. Histological analysis has shown what appears to be a normal cellular response with the accumulation of neutrophils, T cells, B cells and dendritic cells (DC) underlying and adjacent to ORFV infected epidermal cells [19,25,26,27,28]. The numbers of these cells have been observed to increase or decline with the presence of virus with CD4+ T cells being the predominant T cells present in infected skin [6].

The dynamics of the local immune response to ORFV infection have been studied by examining the cells and soluble mediators in afferent and efferent lymph draining the site of infection. These studies have shown that the local immune response to ORFV in reinfected sheep was a biphasic lymph cell response in which CD4+ T cells, CD8+ T cells, B cells and DC were detected (reviewed in [6]). It was found that CD4+ T cells were the most numerous lymphocyte sub-set present in afferent lymph and their numbers peaked on days 4 and 12 post-infection in reinfected sheep. Furthermore a similar pattern was also observed in the production of granulocyte-macrophage colony-stimulating factor (GM-CSF), interleukin-1 (IL-1), IL-8, IL-2 and interferon-γ (IFN-γ) in cultured lymph cells from afferent lymph. Significantly, reinfected animals produce a strong delayed-type hypersensitivity response, indicative of a memory response, when given inactivated ORFV [6,29]. In addition, cytokine analysis of ORFV-infected tissue has shown that the immune response is predominantly a T-helper type 1 response, with epidermal cells, vascular endothelium and uncharacterised cells with lymphocyte morphology producing tumor necrosis factor-α (TNF-α) [28]. These cells increased rapidly in skin during infection. Cells with lymphocyte morphology were shown to express IFN-γ mRNA but only after reinfection. CD4+ T cells were shown to have a critical role in the clearance of the virus. The depletion of CD4+ T cells was associated with increased lesion size and time to resolution and a similar but smaller effect was seen for CD8+ T cell depletion [30]. In addition when sheep were treated with the immunosuppressive drug cyclosporin-A severe ORFV lesions developed and this was associated with the inhibition of IL-2 and IFN-γ in skin [31].

Studies on immunity to ORFV infection in sheep have shown that animals produce a vigorous inflammatory response during the early stage of infection and that the adaptive response appears normal with animals displaying a typical antiviral T-helper type 1 immune response. The ability of ORFV to reinfect its host does not appear to involve impaired memory as a strong delayed-type hypersensitivity reaction to ORFV antigen is observed. The discovery of immunomodulators produced by ORFV may explain how ORFV is able to escape host immunity at least temporarily.

## 4. Orf Virus Genome

The ORFV genome is the smallest within the *Chordopoxvirus* subfamily with a size of 138 kbp [32,33]. In contrast to most other poxviruses, the genome is G+C rich with an overall GC content of 66% [4]. Restriction endonuclease cleavage analyses of ORFV, BPSV and PCPV genomes showed marked variability, although DNA/DNA hybridisation revealed strong interspecies homology between regions in the central core. There was a lack of cross-hybridisation between the terminal fragments suggesting significant differences in the parapoxviruses within this region [34,35]. Genetic studies of the parapoxviruses began in the late 1980s and early 1990s with the cloning of genomic restriction endonuclease fragments of the ORFV strain NZ2 [36,37] and sequencing of specific regions [38,39,40,41,42,43,44]. These studies revealed a remarkable similarity in genetic structure between ORFV and vaccinia virus (VACV) suggesting that they are essentially co-linear within the central region of their genomes [45]. In addition ORFV transcriptional promoter sequences closely matched VACV and the VACV RNA polymerase was shown to recognise the promoters of ORFV genes [39,42,45]. Their transcriptional machinery were also found to be highly conserved where transcription stop sequences, T5NT, are located at the ends ORFV early genes [39,43]. Late gene promoter sequences are also conserved [45].

The complete genome sequences for three ORFV strains and one BPSV strain were published in 2004 and 2006 [32,33]. The complete genome sequence of PCPV was published in 2010-11 [46,47]. These analyses revealed that ORFV isolates from a kid in Texas (OV-SA00) and a lamb in Iowa (IA82) each encoded a 130 putative genes [32] while bioinformatics analysis of a sheep isolate from New Zealand (strain NZ2) showed that there may be an additional two putative genes (ORFs 112.5 and 107.5) in all three isolates [33]. The complete sequence of the ORFV genome confirmed the prediction of previous studies that ORFV shares a large number of its genes with VACV within the central core of the genome [45,48]. The central core region contains homologues of conserved poxvirus genes involved in basic replicative mechanisms, structure, and morphogenesis and these genes are conserved in relative position spacing and orientation, however, there were some differences noted. The homologues of the VACV F9L and F10L genes that are located at the left end of the conserved core in most chordopoxviruses are located at the right end of ORFV, BPSV and PCPV. Notable however is that a number of genes, whose products associated with virion membranes, are lacking in ORFV, BPSV and PCPV.

Phylogenetic analyses of ORFV isolates from different countries around the world have given some indications of possible geographical clustering of strains [21,49,50,51,52,53]. However any conclusions about the global evolution of ORFV must at this stage be interpreted cautiously since the analyses have typically made use of only small numbers of isolates and analysed only a small fraction of the viral genome.

## 5. Orf Virus Virion Structure: Envelope-Membrane Proteins

Few studies have examined the structure of the ORFV virion in any detail. As described above, the complete sequence of ORFV revealed that it has homologues of most VACV structural proteins, suggesting that the core structure of the virions are similar, however, there are a number of differences in proteins that are incorporated into the virion membranes that suggest possible differences in their morphogenesis, intracellular movement, cell-cell transmission and entry [54].

The predominant infectious form of VACV, the mature virion (MV), has a membrane that is derived from the endoplasmic reticulum (reviewed in [55]). In addition wrapped virions are (WVs) are produced in which two additional membranes derived from the *trans*-golgi network, wrap the MV particle ([56,57]. The outermost membrane of this form is lost during egress to produce the extracellular virion (EV) [58]. In VACV specific structural proteins are associated with the envelope-membranes of each form. ORFV has homologues of all the VACV-encoded MV envelope-associated proteins except D8L, but only some of the VACV proteins associated with WV and EV that include A33R, A34R F12L F13L. ORFV does not have homologues of VACV A36R, A56R, B5R and K2 that are associated with either WV or EV. Immunogold labelling of predicted ORFV envelope proteins has provided evidence of MV and WV particles [54]. The ORFV encoded proteins 10 kDa and F1L are homologues of the VACV proteins A27L and H3L that are associated with MV. Immunogold labelling of the 10 kDa and F1L proteins demonstrated that these proteins were associated with ORFV particles isolated from infected lysed cells. Significantly, immunogold labelled ORF-110, a homologue of VACV A34R, could not be detected on particles isolated from lysed infected cells but could be detected on the surface of virus particles in the cell culture medium, suggesting that like VACV, ORFV during egress from the cell looses its outmost membrane exposing this protein on the surface of the EV form. ORFV particles resembling VACV MV and WV particles have been described in ultrathin sections of infected cells [59] also suggesting that such particles exist for ORFV. ORFV has never been observed to bud [60] supporting the view that that the mechanism of egress from infected cells is through fusion with the plasma membrane.

The findings of Tan *et al.* [54] showed that ORFV has wrapped particles of MV, despite the lack of a homologue of VACV B5R, which has multiple functions including a role in membrane wrapping of MV. However ORFV has homologues of other VACV genes known to be involved in wrapping that include F13L and A27L [61,62] suggesting that the mechanism of wrapping could be different to that of VACV. Furthermore ORFV lacks A36R, A56R and K2L that are associated with either the WV or EV envelope. B5R and F13L are involved in the movement of WV on microtubules to the cell periphery [63,64] whilst A36R is critical for the movement of VACV from cell to cell by actin tail formation but not required for EV production [65]. The lack of a homologue of A36R may explain why actin tail formation has not been observed for ORFV. A34R and B5R disrupt the EV envelope prior to fusion during entry [66], whilst A56R and K2L interact with A16L and G9R to prevent fusion of infected cells [67]. The above findings suggest that ORFV has evolved other mechanisms that allow intracellular movement and entry into neighbouring cells or that these mechanisms are not required due to the manner in which ORFV infects keratinocytes and induces their proliferation.

## 6. Orf Virus Genes Involved in Pathogenesis and Virulence

The terminal genomic regions of ORFV represent approximately 20% of the genome and encode factors that determine host range, pathogenesis and virulence [32,33]. The virulence factors identified include an IL-10-like gene [40], CBP [68], VEGF [44], GIF [69], apoptosis inhibitor [70], IFN resistance gene [71,72] and inhibitors of NF-κB [73,74,75]. Like other poxviruses many of the genes within the termini are non-essential and such genes have been identified by genomic sequence analysis of tissue-passaged strains that have undergone spontaneous terminal rearrangements [76,77] as well as the construction of single gene knock-out recombinants [73,74,75,78,79]. Also notable is that BPSV, ORFV and PCPV genomes contain 127 genes with the same relative order and orientation. Bioinformatics analyses have revealed that 15 of the genes found in ORFV, BPSV and PCPV are unique to the parapoxviruses [32,46]. In total, ORFV has 17 ORFs that have no significant homology to genes from other poxvirus genera and 111 genes with homology to genes from other poxvirus genera. This includes 88 of 90 genes conserved within all other chordopoxviruses. Parapoxviruses are unique within the *Chordopoxvirus* subfamily in that they lack homologues of VACV F15L, a gene of unknown function and VACV D9R, a gene encoding a putative nucleoside triphosphate pyrophosphohydrolase [32].

Sequence analysis of the ORFV, BPSV and PCPV genomes revealed that these viruses share a number of features with *Molluscum contagiosum virus* (MOCV) [32,46]. MOCV causes a common wart-like skin infection and has a genome of 188 kbp [80,81], which like parapoxviruses is GC rich. MOCV lacks many genes found in ORFV in particular genes containing ankyrin repeat sequences however it does share several genes that are not found in other poxvirus genera. ORFV 014, 015, 029 are putative orthologues of MC026L, 027L and 043L respectively based on amino acid identity and genomic location [32] and recent studies suggest that they may have roles in viral replication in skin cells. ORFV and MOCV both lack genes present or conserved in other poxviruses. These include homologues of most poxviral genes involved in nucleotide metabolism including homologues of orthopoxvirus ribonucleotide reductase, thymidine kinase, guanylate kinase, thymidylate kinase and a putative ribonucleotide reductase cofactor [32]. Parapoxviruses and MOCV are the only chordopoxviruses lacking homologues of VACV B1R a serine threonine protein kinase. Also absent in parapoxviruses and MOCV are serine protease inhibitor and kelch-like gene families present in other chordopoxviruses. These genes are associated with virulence [82] and are known to affect inflammation, apoptosis, complement activation and coagulation [83]. Delhon *et al.* [32] suggests that the lack of chordopoxvirus-like genes in parapoxviruses and MOCV may reflect adaptation for specific tissue tropism since they appear to replicate in cycling epidermal cells.

## 7. Inhibition of Apoptosis

An anti-apoptotic factor of ORFV encoded by gene *ORFV*125 has been shown to have Bcl-2-like properties [70]. A homologue of this gene has not been found in other viruses but orthologues exist in other members of the *Parapoxvirus* genus.

Apoptotic cell death or cell suicide forms an important host defence mechanism to limit virus replication. Apoptosis can be induced by a variety of extracellular inducers including TNF, apoptosis stimulating fragment ligand (FASL), IFN, natural killer (NK) cells and cytotoxic T lymphocytes as well as agents such as UV light, serum growth factor deprivation and hypoxia and within the cell by macromolecular synthesis of molecules such as viral dsRNA. Viruses have evolved an impressive range of modulators that block apoptosis by extrinsic factors that induce apoptosis via cell-surface death receptors or by intrinsic factors such as DNA damage, endoplasmic reticulum stress or both [84]. The induction of apoptosis by extrinsic or intrinsic pathways results in the activation of caspases that subsequently cleave a large number of cellular proteins leading to cell death. Many large DNA viruses encode Bcl-2-like proteins that counteract the induction of apoptosis [85,86].

The ORFV125 protein is directed to the mitochondria and blocks the release of cytochrome C that would otherwise lead to caspase activation and DNA fragmentation [70]. It was shown that the ability of ORFV125 to inhibit UV-induced apoptosis was comparable to that of the cellular anti-apoptotic factor Bcl-2. It was able to entirely block UV induced activation of the pro-apoptotic Bcl-2 family members Bak and Bax. Although the overall amino acid identity of ORFV125 and Bcl-2 is only 10%, the alignment of ORFV125 and three anti-apoptotic members of the Bcl-2 family revealed that ORFV125 shares predicted structural features and key functional residues with Bcl-2 proteins including BH domains 1 and 3, as well as partial evidence of BH2 and BH4 domains [70].

Mitochondrial apoptosis is regulated specifically by members of the Bcl-2 family. This protein family consists of one class of anti-apoptotic (Bcl-2-like) and two classes of pro-apoptotic (Bax-like and BH3-only) proteins that share 1–4 conserved Bcl-2 homology domains [87]. The pro-apoptotic BH3-only proteins are the initiators of mitochondrial apoptosis. They activate Bak and Bax that are considered to be the executioners of the mitochondrial pathway of apoptosis. Upon activation they permeabilize the mitochondrial outer membrane resulting in a release of pro-apoptotic substances that trigger caspase activation and apoptosis [88,89]. ORFV125 interacts with a range of BH3 only initiators (BimS, Bik, Puma and DP5) thus preventing them from activating Bax and Bak [90]. In addition ORFV125 can inhibit the apoptotic activity of Bax by directly binding to its activated form.

The Bcl-2-like properties of ORFV125, places it in a cluster of poxviral Bcl-2-like factors that include VACV F1L, N1L, M11L and FWPV039 that all possess a mitochondrial-targeting motif. Functional analysis of these proteins, have shown that they too inhibit the mitochondrial pathway of apoptosis [91,92,93,94] and bind to pro-apoptotic Bcl-2 family members. Similar genes have been identified by bioinformatics analyses within each genus of the vertebrate poxvirus subfamily, apart from MOCV. The binding profile of ORFV125 appears to differ from other poxviral Bcl-2-like proteins. While VACV F1L, N1L, M11L and FWPV039 bind to some BH3-only proteins, their main target seems to be Bax-like proteins. All four have been shown to interact with Bak and activated Bax [92,94,95,96,97,98,99,100]. ORFV125’s failure to bind to Bak but its ability to bind a wide range of BH3-only proteins clearly distinguishes it from other poxvirus relatives [90]. A further overall difference is that other poxviruses commonly encode multiple inhibitors of apoptosis that interfere with death receptor pathways or inhibit caspases [101] whereas no other anti-apoptotic factors have currently been identified for ORFV.

## 8. Inhibitors of the Nuclear Factor-κB Signalling Pathway

ORFV has evolved novel strategies to modulate the host cell responses regulated by the nuclear factor-κB (NF-κB) signalling pathway. Three factors encoded by ORFV have been described by Diel *et al.* [73,74,75].

NF-κB mediates expression of a wide range of cellular genes which are critical for early antiviral responses and modulate innate immunity, inflammation and apoptosis [102]. Furthermore, the NF-κB signalling pathway plays a vital role in the regulation of innate immune responses and the maintenance of skin homeostasis by regulating keratinocyte proliferation, differentiation and apoptosis [103]. Various stimuli including proinflammatory cytokines TNF-α and IL-1 and virus products such as capsid proteins and nucleic acids lead to the phosphorylation of inhibitor-kappa B (IκB) proteins by IκB kinases (IKK complex), resulting in proteasomal degradation of IκB and nuclear translocation of NF-κB subunits [104]. Not surprisingly poxviruses have evolved various strategies to inhibit NF-κB given its central role in innate immunity in skin. Viral encoded proteins that inhibit NF-κB have been described for members of the *Orthopoxvirus*, *Leporipoxvirus*, *Yatapoxvirus* and *Molluscipoxvirus* genera [102].

Homologues of known poxviral NF-κB inhibitors are absent from parapoxviruses and identification of such genes involved preliminary screening by transcriptional profiling and microarray analysis of cells infected with various knock-out viruses. Expression of specific genes by transient transfection was used to determine cellular location, effects on NF-κB signalling and specific interactions with NF-κB signalling factors.

*ORF024* is transcribed early and encodes a protein that localises to the cell cytoplasm. ORFV024 significantly decreased TNF-α induced phosphorylation and nuclear translocation of NF-κB-p65, phosphorylation and degradation of IκBα, and phosphorylation of IκB kinase (IKK) subunits IKKα and IKKβ [74]. IKK activation is the bottleneck for most NF-κB activating stimuli and ORFV has evolved a strategy to target this signalling pathway at its most critical point. Experimental infection of lambs showed that deletion of *ORFV024* had no significant effect in disease severity. ORFV024 homologues share over 90% sequence identity among ORFV strains but lower identity with homologues from BPSV strains (62%).

*ORFV002* is an early-late gene and encodes a protein that localises to the nucleus. ORFV002 suppresses NF-κB mediated gene expression induced by TNF-α and LPS [75]. It doesn’t affect the translocation of NF-κB but was shown to decrease acetylation of NF-κBp65 by interfering with its association with p300. Deletion of *ORFV002* did not affect the virulence of ORFV in its natural host. *ORFV002* is conserved in PCPV but notably is lacking in BPSV.

*ORFV121* is an early late gene and encodes a protein that localises to the cytoplasm. ORFV121 binds to and inhibits the phosphorylation and nuclear translocation of NF-kB-p65 [73]. In contrast to the other ORFV NF-κB modulators identified, deletion of *ORFV121* markedly decreased ORFV virulence and pathogenesis in sheep. A PCPV homologue lacks most of the amino-terminal region of ORFV121 [73]. The homologue in BPSV strain AR02 has only 39% identity with ORFV121.

The lack of homologues of other known poxvirus NF-κB signalling inhibitors in members of *Parapoxvirus* genus further suggests significant evolutionary divergence. Notably, within the *Parapoxvirus* genus, there appears to be considerable sequence diversity within the putative NF-κB inhibitory genes identified.

## 9. Inhibition of Interferon

A homologue of the VACV IFN resistance gene E3L has been described in ORFV [71]. *ORFV020* is an early gene that is located at the left end of the ORFV genome. The amino acid sequence of ORFV020 shares 31% amino acid identity (57% similarity) with E3L. *ORFV020* is highly conserved in ORFV strains with their predicted amino acid sequences varying by approximately 3%. The recombinant protein expressed as a fusion protein in *E. coli*, bound double stranded RNA but not double stranded DNA or single stranded RNA. ORFV020, expressed as a thioredoxin fusion protein, inhibited the activation of IFN-inducible dsRNA-dependent kinase of sheep [72]. PKR is involved in the inhibition of protein synthesis as part of the anti-viral state in infected cells. Furthermore ORFV020 expressed transiently in interferon-treated ovine fibroblasts protected Semiliki Forest virus from the antiviral effects of both type I and type II IFNs.

Studies have been conducted to determine if *ORFV020* complements the deletion of the VACV E3L gene [105]. A recombinant VACV expressing the ORFV020 gene was indistinguishable from *wt* VACV in cell culture but was over a 1000-fold less pathogenic than *wt* VACV following intranasal infection of mice. These results suggest the specific adaptation of the ORFV020 gene to its natural host.

## 10. Interleukin-10-Like Factor: Suppression of Inflammation and the Adaptive Responses

A poxvirus IL-10-like gene was first reported in ORFV and shares remarkable similarity to mammalian IL-10 [40]. ORFV-IL-10 is an early gene that is located at the right end of the genome. IL-10-like genes have only been found in other parapoxviruses including BPSV [32], PCPV [46] and PVNZ (unpublished) although homologues of IL-24, which is a member of the IL-10 family [106], have been described for yatapoxviruses (Yaba-like disease virus (YLDV) [107] and capripoxviruses (*Lump skin disease virus*, *Goatpox virus* and *Sheeppox virus*) [82]. Mammalian IL-10 is a multifunctional cytokine that has suppressive effects on inflammation, antiviral responses and T-helper type 1 effector function, MHC class II antigens and co-stimulatory molecules on macrophages [108]. In addition it enhances B cell survival and proliferation. IL-10 can also block NF-κB activity and has a role in regulating the JAK-STAT signalling pathway.

The IL-10-like virokine encoded by ORFV_NZ2_ shows high levels of identity to the IL-10 of sheep (80%), cattle (75%), humans (67%) and mice (64%) as well as to the IL-10-like genes of Epstein-Barr virus (EBV) (63%) and equine herpes virus (67%) [40]. This identity is highest over the final two-thirds of the viral protein and greater than 98% identical with ovine IL-10 within this region. The relatedness of the ORFV-10 to ovine IL-10 is less apparent at the DNA level reflecting different codon usage by ORFV genes in general.

The functional characterisation of ORFV-IL-10 has revealed that it has all the activities of mammalian IL-10 and is functionally indistinguishable from ovine IL-10. These activities are in contrast to EBV IL-10 that displays only a subset of activities of human IL-10. The activity of ORFV-IL-10 clearly differs from EBV IL-10 in its ability to stimulate murine thymocyte proliferation [40] and co-stimulate the proliferation of murine mast cells (MC/9) [109]. ORFV-IL-10 was equally as effective as ovine IL-10 at inhibiting the production of TNF-α from activated murine peritoneal macrophages [109], TNF-α and IL-8 production from stimulated ovine macrophages and keratinocytes and IFN-γ and GM-CSF from peripheral blood lymphocytes [110]. ORFV-IL-10 was also shown to have equivalent immunosuppressive activity to human IL-10 [111]. ORFV-IL-10 is a potent anti-inflammatory virokine and deletion of this gene severely attenuates the virus [78].

The most likely explanation for the different activities displayed by ORFV-IL-10 and EBV-IL-10 involves their interaction with the IL-10 receptor. The functional receptor complex of IL-10 consists of two identical subunits, IL-10R1 and IL-10R2 [112,113] and signal transduction can only occur in cells expressing both subunits [113]. Analysis of the crystal structure of the hIL-10-IL-10R1 complex has shown that the IL-10 dimer binds symmetrically to two soluble IL-10R1 chains [114]. The structure of the human IL-10-IL-10R1 complex interacting with two IL-10R2 chains has been predicted [115,116]. The amino acids of human IL-10 that interact with both IL-10R1 and IL-10R2 are located between residue 10 (Phe) of the mature polypeptide to the C-terminal end of the molecule. Based on the human IL-10/IL-10 receptor interaction, examination of the ORFV-IL-10 and ovine IL-10 polypeptide sequence shows that the equivalent amino acids of human IL-10 that are known to interact with IL-10R1 and IL-10R2 are identical between ORFV-IL-10 and ovine IL-10. In the case of EBV IL-10 there are a number of differences in amino acids predicted to contact human IL-R2 and these differences likely explain its lack of stimulatory activities. It is also apparent that BPSV-IL-10 has evolved in its host, as that region of the molecule that is predicted to interact with the receptor is also identical to bovine IL-10. It is clear that although at the nucleotide level, ORFV-IL-10 has undergone significant evolutionary change compared to ovine IL-10 (synonomous substitutions), it has largely retained the polypeptide structure of its ovine counterpart and biological activities of ovine IL-10. It is not currently known why the N-terminal region of ORFV IL-10 is so different from its mammalian counterpart where numerous non-synonmous substitutions have taken place.

The fact that the poxvirus IL-10 gene is only found in parapoxviruses and within approximately the same location of the genome suggests that it represents a recent acquisition by horizontal transfer from mammals that has undergone change as parapoxvirus species have evolved and adapted to different hosts. Alternatively, the IL-10 gene may have been captured independently by BPSV and ORFV from their host species [117].

## 11. Chemokine Binding Protein: Inhibition of Immune Cell Trafficking

ORFV produces a soluble secreted CBP [68]. The ORFV CBP gene is located at the right end of the genome and is expressed early. It has no mammalian homologue but CBPs are encoded by other poxviruses and herpes viruses [118].

Chemokines are a large family of secreted chemotactic proteins that activate and regulate inflammation induced leukocyte recruitment to sites of infection as well as homeostatic migration of leukocytes through lymphoid organs [119,120]. Members of the family are classified as CC, CXC, CX3C and C according to the spatial arrangement of cysteine residues within the N-terminus of the molecule.

Discrete and overlapping residues on the surface of chemokines define the specificity for binding and signalling through G protein-coupled receptors [121]. Interaction of chemokines with cell-surface glycosaminoglycans provides a means for chemokines to form solid phase gradients that help guide leukocytes along endothelial surfaces and into tissue [122,123,124].

Viral CBPs have the ability to disrupt chemokine gradients thereby inhibiting the trafficking of immune cells to sites of infection [118,125]. Within the poxvirus family CBPs are produced by the *Parapoxvirus*, *Leporipoxvirus* and *Orthopoxvirus* genera. The CBPs of leporipoxviruses and orthopoxviruses are specific for CC chemokines whereas ORFV-CBP binds across a broad spectrum of chemokines including members in the CC, CXC and C classes [68,126,127].

ORFV CBPs share low identity to the CBP-II family of proteins encoded by leporipoxviruses and orthopoxviruses [68]. The alignment of ORFV NZ2 and NZ7 CBPs shows only 12%–18% identity and 26%–32% similarity with poxvirus CBP-II proteins [68]. Despite the overall low identity of the CBP-II proteins with ORFV CBPs they share regions of identity and similarity across their entire sequence.

The ORFV-CBP interacts with a range of proinflammatory CC chemokines that include eotaxin (CCL11), MCP-3 (CCL7), MIP-1α (CCL3), MIP-1β (CCL4), and MCP-1 (CCL12) with high affinity (Kd < 1) [68] as well as the constitutive chemokines CCL19 and CCL21 [127]. Two CC chemokines it does not bind to are monocyte-derived chemokine (CCL22) and thymus and activation regulated chemokine (CCL17). ORFV CBP also binds with high affinity to lymphotactin (XCL1) [68] and members of the CXC class of chemokines, CXCL2 and CXCL4 (unpublished data). The screening of various chemokine families by surface plasmon resonance indicates that ORFV-CBP possesses a binding specificity and affinity similar to that of the CBP-II family but it also has the capacity to bind XCL1 and CXCL2 and CXCL4.

ORFV-CBP has been demonstrated to be a potent inhibitor of immune cell trafficking in mouse skin inflammation models. The predominant chemokines produced during damage and inflammation of epithelial tissues are CCL2, CCL3 and CCL5 [128,129].

These chemokines can be induced by injection of small amounts of lipopolysaccharide (LPS) intradermally into mouse skin [130,131]. Nanogram quantities of purified CBP co-injected with LPS potently inhibited the recruitment of both Gr-1+/CD11b+ monocytes [126] and CD11c+/MHC-II+ dendritic cells [127]. Furthermore CBP inhibited the migration of *ex vivo* CpG-activated DC to inguinal lymph nodes and prevented T cell activation, a process in which DC trafficking is critically dependant on the constitutive chemokines CCL19 and CCL21. Our findings from the mouse skin inflammation model show that ORFV-CBP injected as a purified protein can inhibit inflammatory cell recruitment from blood to the skin and prevent activation of T cells in peripheral lymph nodes by antigen presenting cells. In the context of virus infection, ORFV-CBP was shown to be a potent virulence factor (S Fleming, unpublished data).

Detailed molecular interaction of CBP with chemokines has been studied. The ORFV-CBP, in common with CBP-II family of proteins contacts residues on chemokines that overlap with those residues used by the chemokine to bind the receptor [68]. Using amino acid point mutants of MCP-1 and surface plasmon resonance at least four residues were identified that are critical in binding to ORFV-CBP. Moreover these residues overlap with residues of MCP-1 that contribute to the recognition by CCR2b [132]. The results provided a structural basis for the ability of ORFV-CBP to block CC chemokines from binding their cognate receptors. It was concluded from these studies that the ORFV-CBP binding occludes the receptor-binding site of chemokines in a manner similar to that of the CBP-II family of chemokine inhibitors.

The sequence and structural similarities between the ORFV-CBP compared with the CBP-II family strongly suggest that these proteins derive from a common protein ancestor CBP [68]. Furthermore, it is thought that the close sequence relationship with GIF provides an evolutionary link that bridges the CBP-II proteins of *Leporipoxvirus* and *Orthopoxvirus* genera with GIF which may have been generated from a duplication of the ORFV CBP gene early after the divergence of the *Parapoxvirus* genus. It would appear that during the course of evolution ORFV-CBP has lost two cysteine residues compared with the CBP-II family. The absent disulphide bond may provide additional conformation flexibility to accommodate a broader range of chemokine binding than the CBP-II members. ORFV-CBP is conserved in other *Parapoxvirus* genera but with low identity and has less than 40% identity to BPSV-CBP although both have similar binding specificities across three classes of chemokine (unpublished data).

## 12. Inhibitor of Granulocyte-Macrophage Colony-Stimulating Factor and Interleukin-2

ORFV encodes a novel soluble secreted protein inhibitor of ovine granulocyte-macrophage colony-stimulating factor (GM-CSF) and IL-2 known as GIF [69]. GIF is the only known viral or cellular protein to bind both GM-CSF and IL-2. The GIF gene is located at the right end of the ORFV genome and is expressed at intermediate/late times after infection. In solution GIF forms homodimers and tetramers and binds ovine GM-CSF and ovine IL-2 with high affinity but not their human counterparts. Characterisation of the functional activities of GIF demonstrated that it inhibited the hematopoietic activity of ovine GM-CSF in a soft agar bone marrow cell colony assay and the biological activity of ovine IL-2 in a T cell proliferation assay [69].

GM-CSF is produced by a number of cell types including T cells. It stimulates neutrophil, monocyte and eosinophil myelopoiesis and the recruitment or activation of these cells in tissues [133]. GM-CSF also regulates the differentiation and function of dendritic cells. IL-2 is a multifunction cytokine that is produced by T cells and stimulates T cell and NK cell activation and proliferation and B cell proliferation [134].

The role of GIF in ORFV infection is not known, but studies on the localised immune response at the site of infection may suggest a role in subverting the adaptive responses. Both ovine GM-CSF and IL-2 have been detected in afferent and efferent lymph after ORFV reinfection of sheep [135,136] and the main source of these cytokines is the CD4+ T cell. These cells have been shown to accumulate in large numbers adjacent to and underlying ORFV-infected cells in the epidermis. Furthermore IL-2 and IFN-γ have been implicated in protective immunity to ORFV reinfection [30,31,135,137]. The role of GIF in inhibiting the activity of GM-CSF is less certain. GM-CSF is involved in the activation of macrophages and neutrophils, which are both seen at the site of ORFV infection but also GM-CSF regulates antigen presentation by dendritic cells. In addition GM-CSF and TNF-α have been shown to be involved in the recruitment of dendritic cells to ovine dermis and in supporting the survival and proliferation of afferent lymph veiled dendritic cells in culture [138]. It is thought that the modulation of GM-CSF in the vicinity of ORFV infected keratinocytes could affect dendritic cell function.

The GIF gene sequence is also present in PCPV and BPSV [139]. The predicted amino acid sequences of the PCPV and BPSV proteins share 88% and 37% identity with ORFV-GIF respectively and both retain the 6 cysteine residues and the WSXWS box-like motif that are required for biological activity. However, functional analyses of the two proteins showed that while PCPV-GIF bound bovine IL-2 and bovine GM-CSF, surprisingly the BPSV homologue bound neither [139].

Bioinformatics analyses have attempted to identify the origins of the GIF gene [69,139,140]. The only other known viral protein to bind IL-2 is a secreted 38 kD protein encoded by *Tanapox virus*. Although this protein binds human IL-2, IL-5 and IFN-γ [141] it does not show any resemblance to GIF. It does not appear that GIF is derived from a cytokine receptor, as there have been no regions of homology found with cytokine receptors [69]. However it was found that many of the biochemical properties of mammalian GM-CSF receptors that are required for efficient binding of GM-CSF are also critical for GIF binding to ovine GM-CSF [140]. Site directed mutagenesis of GIF demonstrated that a sequence motif (WDPWV) related to the WSXWS motif of the type 1 cytokine receptor superfamily was necessary for biological activity [140]. It was also noted that GIF could be related to the VACV A41-like family of proteins that have some sequence homology to the VACV CC chemokine inhibitory (CCI) protein. As described above it seems likely that GIF is derived from a common poxvirus ancestral CBP gene and has evolved its unique binding specificities in its natural host, sheep.

## 13. Homologue of Vascular Endothelial Growth Factor: Increasing Cellular Substrates for Viral Replication

ORFV was the first virus reported to encode a protein with homology to mammalian vascular endothelial growth factor (VEGF). The ORFV VEGF gene is transcribed early during infection from a gene located adjacent to the inverted repeat at the right end of the genome [44]. Subsequent to the discovery of the VEGF gene in ORFV, homologues were identified near the right terminus of PCPV [142] and PVNZ [143], and the left terminus of BPSV [32,144]. VEGF genes have not been found in any other poxviruses and to date the only other viruses reported to encode VEGF genes are members of the *Megalocytivirus* genus within the *Iridovirus* family, which infect a range of fish [145,146,147].

Members of the mammalian family of VEGFs are critical regulators of new blood vessel formation during embryogenesis and in the adult during processes such as wound healing [148]. The VEGF family, which comprises of VEGF-A, VEGF-B, VEGF-C, VEGF-D and placental growth factor (PlGF), interact with one or more of the high affinity VEGF receptors (VEGFRs), VEGFR-1, VEGFR-2 or VEGFR-3 [149]. In addition, VEGFs can bind two co-receptors, neuropilin (NRP)-1 and NRP-2 [150]. VEGFs promote angiogenesis by stimulating endothelial cell proliferation, migration and survival, and promote vascular permeability, primarily through VEGFR-2. VEGFR-1 plays a role in endothelial cell and monocyte migration and inflammatory cytokine production, but also acts as a ligand-binding molecule that by sequestering VEGFs regulates VEGFR-2 signalling. A subset of the VEGFs also promotes lymphangiogenesis through their interaction with VEGFR-3 [151].

The purified ORFV VEGF directly induces endothelial cell proliferation, vascular permeability and angiogenesis in skin [152,153]. These biological functions are mediated exclusively via its interaction with VEGFR-2, as the ORFV VEGF does not bind VEGFR-1 or VEGFR-3 [152,154,155]. This receptor-binding spectrum differs from that of any mammalian VEGF but is, in the most part, conserved by the other parapoxvirus VEGFs [142,143,144]. This discovery led to the classification of the parapoxvirus VEGFs as a new subgroup within the VEGF family, collectively designated VEGF-E.

The use of the generic term VEGF-E, however, under-represents the divergence between the parapoxvirus VEGFs. Only 41-61% amino acid identity is shared between VEGF variants from different species of parapoxvirus, which is on par with 25-51% identity the parapoxvirus VEGFs share with VEGF-A [32,44,142,143,144]. Genetic analysis of different strains of ORFV has revealed further sequence disparity, with two individual isolates, ORFV_NZ2_ and ORFV_NZ7_ showing only 41% identity to each other [44,156]. Most ORFV strains have been shown to carry ORFV_NZ2_-like versions of the VEGF gene but their predicted amino acid sequences vary by up to 31% [33,156,157,158,159,160,161]. Although the parapoxvirus VEGFs bind VEGFR-2 and are mitogens for endothelial cells, they in fact differ in their affinities for VEGFR-1 and NRP-1 and their abilities to induce monocyte chemotaxis and vascular permeability [144,153]. The VEGF from BPSV was also functionally more similar to VEGF-A, as it showed significant binding to VEGFR-1 and induced monocyte migration [144]. The VEGFs from ORFV_NZ7_ failed to induce vascular permeability while only the VEGF from ORFV_NZ2_ showed detectable binding to NRP-1 [153,162,163].

Despite low sequence homology, the crystal structure of the ORFV_NZ2_VEGF revealed high similarity to VEGF-A [164]. Distinct conformational differences were however observed in loop L1 and particularly in L3, which contains a highly flexible glycine-serine-rich motif that differs from other VEGFs. Mutational analyses have shown that these loop regions are critical to the VEGFR-2 selectivity of the viral VEGF [164,165]. Additional domains, namely a receptor-linker groove and *O*-glycosylated C-terminus, appear to regulate the interaction of the viral VEGF with VEGFR-1 and NRP-1 [156,164,166,167]. Structural predictions and molecular characterisation of the other parapoxvirus VEGFs suggest that while they conserve the characteristic VEGF homodimeric structure and cysteine knot motif, minor differences in the viral-specific VEGF loop extension and *O*-glycosylated C-terminus may influence their receptor interactions [142,143,144]. In addition, specific residues of BPSV VEGF, outside the known receptor-binding face of the VEGFs, are predicted to open the receptor-binding face and linker groove, potentially enhancing its ability to bind VEGFR-1 [144].

The extent of sequence variation observed between VEGFs from different isolates of ORFV, and other parapoxviruses, is not a common feature of poxviruses and its evolutionary significance is unclear [117]. The lower G+C content of the viral VEGF genes, compared with the content of the flanking sequences suggests a relatively recent acquisition by the virus of the VEGF gene from a mammalian host [32,44]. Genetic drift however does not account for the variation between ORFV_NZ2_-like variants, which is similar to that between homologous proteins of different species, or the variation between VEGFs of parapoxvirus species, and is more akin to the variation between homologous proteins of different genera of the same family [117,156]. It has been proposed that the sequence divergence may have been generated by selection against VEGFR-1 binding and its associated recruitment and activation of cells that contribute to the host’s anti-viral responses [156,167]. However, the extent of the sequence differences between isolates and species suggests that recombination may have occurred during co-infection of a host. In addition, rearrangements may have led to the VEGF genes occupying a different position in the genome of BPSV compared with other parapoxviruses [117,156].

**Figure 2:**
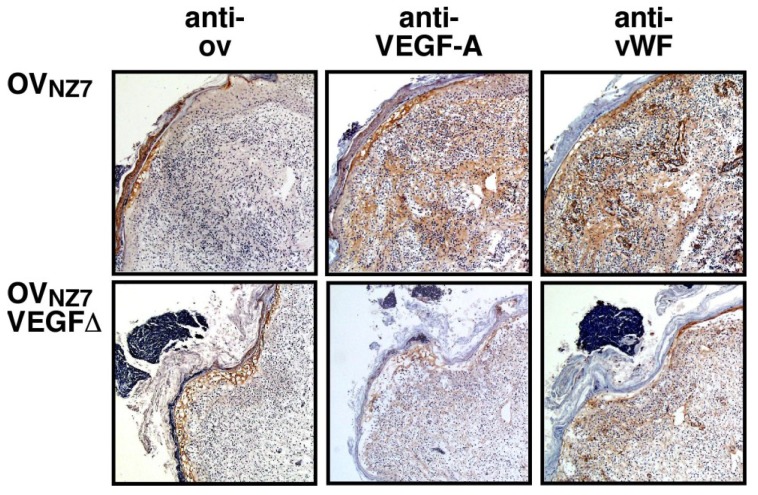
Orf virus (ORFV) vascular endothelial growth factor (VEGF) promotes blood vessel growth in ORFV infected sheep. Scarified skin was inoculated with *wt* ORFV_NZ7_ or ORFV-VEGF∆. Shown is immunohistological stained tissue from lesions of *wt* infected and VEGF∆ infected animals at 6 days post-infection (magnification × 50). The sections were stained with peroxidase conjugated anti-ORFV F1L (major coat envelope protein) antibody (anti-OV), anti-VEGF-A antibody (anti-VEGF-A) and anti-von Willebrand factor antibody (anti-vWF) that stains the endothelial cells of blood vessels. The figure was reproduced from Figure 4 in [173] with permission from Elsevier publishing.

Parapoxvirus lesions have been described as extravagantly proliferative and persistent, and reminiscent of a sustained wound healing response [12,13,168,169,170,171,172]. The viral VEGFs are, at least in part, responsible for the extensive vascular proliferation beneath parapoxvirus lesions (see Figure 2). In the absence of a functional VEGF, the infected ORFV lesions lack the extensive proliferation of blood vessels and dermal swelling associated with wild type infections [79,173]. Intriguingly the characteristic pattern of epidermal hyperplasia and finger-like projections of epidermis deep into the dermis observed in wild-type lesion was also absent with the VEGF deletion mutant [79,173]. The viral VEGF has since been shown to directly stimulate epidermal migration, proliferation and regeneration, partly through the induction of matrix metalloproteinases [174].

Parapoxvirus infections initiate in damaged skin, with the virus replicating in newly dividing epidermal cells that are driven by the host’s wound healing response [168,170]. Expression of a VEGFR-2-selective VEGF may enhance viral growth by maintaining this regenerative response by directly promoting epidermal regeneration and by indirectly supplying the necessary nutrients. In this regard the viral VEGF acts in a similar manner to the epidermal growth factor encoded by most poxviruses but not by parapoxviruses [83,175]. The VEGFs from ORFV, PCPV and PVNZ also appear to have adapted to the cutaneous environment of the muzzle and teats by limiting their ability to bind VEGFR-1 and activate the anti-viral immune response. In contrast, the VEGF encoded by BPSV may have not been subjected to the same selection pressure to lose VEGFR-1 binding as it preferentially infects the mucosal lining of the tongue and mouth [176,177,178], which is a more tolerant immune environment [179,180]. The ability of the viral VEGF to induce epidermal hyperplasia and dermal oedema may also contribute to scab formation, as lesions induced by ORFV in the absence of functional VEGF had essentially no scab [79]. Scab shed from the parapoxviruses contain substantial amounts of infectious virus and the scab provides protection from environmental inactivation for as much as a year after being shed [15].

## 14. Manipulation of Cell Cycle: A Poxvirus Anaphase-Promoting Regulator

An unusual gene present in ORFV, and all parapoxviruses encodes a RING-H2 protein with sequence similarities to subunit 11 of the anaphase-promoting complex (APC) [181]. The APC is a multi-subunit ubiquitin ligase with key roles in cell cycle regulation, controlling both exit from mitosis and the duration of G1 by directing the ubiquitin-proteasome-dependent degradation of a range of proteins [182]. The catalytic core of the complex is formed by subunit 2 (APC2), a scaffold protein, and APC11, a RING-H2 protein. The ORFV homologue of APC11 is encoded by gene ORFV014 and the protein has been called PACR (poxvirus anaphase promoting complex regulator) in light of its apparent ability to manipulate APC activity. Unlike APC11, PACR lacks ubiquitin ligase activity and domain swap experiments have pointed to differences in specific regions of the RING domains of the two proteins as being responsible for this functional difference. It has also been shown that, like APC11, PACR interacts directly with APC2, and that PACR is likely to compete with APC11 for incorporation into APC [183]. Expression of PACR leads to deregulation of the cell cycle and the accumulation of APC substrates, consistent with impaired APC function. Deletion of the PACR gene led to a marked reduction in viral yield and plaque size, demonstrating that PACR is significant in ORFV replication.

Viruses commonly coerce host cells into providing an environment supportive of viral replication and frequently this includes targeting of cell cycle regulation. However, only a modest number of viruses have been shown to target APC and the analysis of PACR’s activities revealed a previously unknown mechanism of viral manipulation of APC [184]. A key function of APC is to maintain cells in G0/G1 and APC must be turned off in order for cells to enter S phase. This suggests a model in which PACR contributes to creating an S phase-like state to support viral replication. Circumstantial evidence provides further support for this model. Most chordopoxviruses do not encode a PACR homologue but they do encode nucleotide metabolism enzymes such as thymidine kinase and ribonucleotide reductase. In contrast ORFV does not encode versions of these nucleotide metabolism enzymes. Intriguingly, cellular versions of these enzymes are ubiquitinated by APC and their levels are kept low in G1 phase, in part by APC activity, whereas expression of these enzymes rises in the G1/S phase transition when APC is inactive. In light of this correlation between the presence of PACR and the absence of thymidine kinase and ribonucleotide reductase genes (and vice-versa), it is tempting to speculate that poxviruses either encode their own nucleotide metabolism enzymes or encode PACR so as to inhibit APC and promote an S phase-like state in which the cell provides these enzymes. This possibility is given further weight when one considers that PACR homologues are encoded by parapoxviruses, MOCV, and *Crocodilepox virus* but none of these viruses encode either a thymidine kinase or a ribonucleotide reductase [181]. An exception to this trend is the unclassified poxvirus, *Squirrel poxvirus* (SQPV), the genome sequence of which was recently lodged in databases (NCBI Reference Sequence: NC_022563.1). SQPV encodes a PACR homologue and consistent with the pattern of other poxviruses it does not encode a ribonucleotide reductase but it does encode a thymidine kinase homologue. Functional analyses of these enzymes are yet to be reported. No functional examinations of the other poxviral versions of PACR have been reported but bioinformatic analyses indicate they share the features of PACR shown to be responsible for its lack of ubiquitin ligase activity and suggest they too are likely to inhibit APC function.

There are other factors that correlate with the presence of PACR. All PACR-encoding poxviruses have G+C-rich genomes whereas all other chordopoxviruses are A+T-rich viruses. It has long been a puzzle as to how the divergence in G+C content across the chordopoxvirus subfamily arose and what selection pressure must operate to maintain it, while conserving the amino acid sequences of 80 or more proteins expressed by all members of the subfamily. Perhaps the presence of PACR in conjunction with the absence of specific nucleotide metabolism genes plays a part in the selection pressure.

Another factor that correlates with the presence of PACR is growth in the epidermis. All PACR-expressing poxviruses grow in the epidermis and, at least for parapoxviruses and MOCV, their growth is strictly limited to these cells. ORFV is detected in zones containing differentiated cells [25], suggesting it is likely to require a means of manipulating cells into a state supportive of viral genome replication. PACR may thus represent an adaption to the specific environment in which ORFV and other PACR-encoding viruses replicate, stimulating differentiated epidermal skin cells, via the functional disruption of APC, to provide cellular factors to assist viral DNA replication. Furthermore, recent data have shown that key metabolic enzymes are targeted by APC [185]. These data link decreased APC activity with the increased metabolic demands arising during cell proliferation, raising the possibility that PACR’s inhibition of APC may affect the provision of cellular resources additional to nucleotides.

A phylogenetic analysis based on the concatenated amino acid sequences of 29 orthologous poxvirus proteins conserved across all established genera of chordopoxviruses, revealed a clade comprised of ORFV, BPSV, MOCV and *Crocodilepox virus* [117]. This clade matches the distribution of PACR homologues among poxviruses (SQPV was not analysed), raising the possibility that poxviral PACRs represent divergence from a single ancestral acquisition event rather than a set of independent acquisitions events. Consideration of this possibility is made more difficult by the absence of available sequence information for the APC11s of either squirrels or Nile crocodiles. However, mammalian APC11s show very high inter-species amino acid sequence identities and even the APC11 of the arboreal lizard, *Anolis carolinenis*, shares 94% amino acid sequence identity with human APC11.

Examination of the inter-relatedness of the viral APC11 homologues shows that mammalian poxvirus PACRs share 35–40% amino acid sequence identity between genera, whereas the crocodilepox virus PACR has 20-29% sequence identity with other PACRs. And all PACRs show between 26-30% amino acid sequence identity with human APC11. Together these observations suggest that PACRs represent divergence from a single ancestral acquisition event. That interpretation further suggests that all PACR-encoding, G+C-rich poxviruses share a common ancestor that arose after divergence from the ancestor of the remainder of the chordopoxviruses. Further detailed phylogenetic analyses will be required to address this possibility.

## 15. Orf Virus Infection of Skin: An Evolutionary Masterpiece of Adaptation

The genes of ORFV that are most distantly related to or absent from other poxviruses are involved in host range, virulence, and pathogenesis. The functional characterisation of these genes illustrates how exquisitely ORFV has adapted, during the course of evolution, to replicate in keratinocytes within the epidermal layer of the skin.

The skin has a number of functions that include forming a physical barrier to the environment, maintaining body temperature and providing a complex immune system to defend the host against infectious pathogens. Keratinocytes form the differentiated layers of cells termed the epidermis that are strategically positioned in the outermost layer of the body. The dermis, which underlies the epidermis is considerably more complex and is composed of a matrix of collagen, elastin and reticular fibres and contains many specialised cells such as various dendritic cell subsets and T cell subsets, natural killer cells, macrophages, mast cells and fibroblasts [1]. The dermis is drained by lymphatic and vascular conduits, through which migrating cells can traffic. While the innate response to skin infection is largely initiated within the epidermis, dendritic cells from the dermis and blood are recruited to the epidermis during skin infections to initiate the adaptive immune responses.

The type of factors that ORFV encodes suggests that it has the ability to subvert the host’s immediate early innate responses. Keratinocytes act as proinflammatory signal transducers responding to non-specific stimuli by secreting inflammatory cytokines, chemotactic factors, antimicrobial peptides and adhesion molecules and IFNs into the epidermal compartment. These factors are produced via the NF-κB signalling pathway, as a result of Toll-like receptor activation [1], inflammasome activation and other sensory molecules that detect foreign nucleic acids and cytokines.

Type 1 IFNs are produced in abundance by activated keratinocytes [186] and orthopoxviruses produce a number of factors that block the production of these molecules [83], however no such factors have been reported for ORFV although it seems likely that such factors are produced. ORFV does produce a homologue of the VACV E3L gene ORFV020 that has been shown to bind dsRNA and thus prevent the activation of the dsRNA-dependent IFN inducible protein kinase (PKR) that blocks viral protein synthesis [71,72]. In addition ORFV has been shown to inhibit the effects of both type 1 and type 2 IFNs [72] although specific genes such as the C7L and K1L genes of VACV have not been identified [187]. Nor is there evidence in ORFV of genes related to genes of other poxviruses with structural and functional similarities to C7L [188].

Inhibition of the NF-κB signalling pathway is a common mechanism used by poxviruses to suppress the induction of inflammatory cytokines [83]. The factors that ORFV produces to inhibit this pathway have functional similarities to other poxvirus NF-κB signalling inhibitors however they are unique and have no homology to these genes [73,74,75]. It’s likely that the ORFV genes are distant relatives of ancient poxvirus genes that have undergone considerable change during the course of evolution. It is possible that ORFV produces further factors that inhibit NF-κB signalling, given the large numbers of such inhibitors produced by other poxviruses, however significantly the ORFV factors discovered to date target NF-κB signalling at its most critical points.

Apoptosis is a highly effective innate response employed by infected cells to inhibit viral replication. Thus far ORFV has only been shown to encode one factor that inhibits the apoptotic response by inhibiting the release of cytochrome C from the mitochondria [70]. There is no evidence that ORFV produces factors that bind caspases or factors that disrupt the death receptor mechanisms induced by TNF and FAS that are employed by other poxviruses [189,190,191]. It’s possible that these factors are not employed in skin. Recent studies have shown that Fas exerts antiapoptotic effects in the epidermis in contact hypersensitivity responses of the skin and in the tissue response of the epidermis to UVB irradiation [192]. In another study it was found that keratinocytes were not susceptible to apoptosis when treated with IFN-γ and TNF-α alone or in combination [193]. Furthermore, serpins have not been found such as CrmA that protects cells from perforin-dependent apoptosis induced by cytotoxic T cells and NK cells [194,195] and it’s possible that these cells do not come into contact with infected keratinocytes [136].

During the initial phase of non-specific cutaneous inflammation, keratinocytes release IL-1β and TNF-α [1,2]. IL-1β and TNF-α activate the dermal vascular endothelium, which upregulates the expression of adhesion molecules involved in the recruitment of leukocyte through the endothelium. In conjunction with chemokines, these cytokines direct the migration of leukocytes from the circulatory system into the epidermis. TNF-α expression by keratinocytes is downregulated by cellular IL-10 during inflammation [196]. This activity suggests that a critical role of ORFV-IL-10 is to dampen the skin inflammatory response by inhibiting the production of proinflammatory cytokines produced by activated keratinocytes and other inflammatory cells during the early stages of skin inflammation. IL-10-like genes are unique to the parapoxvirus genus however a few poxviruses such as *Tanapox virus* produce an IL-24-like cytokine that is closely related to IL-10. Other poxviruses that cause systemic infection have evolved a range of secreted anti-inflammatory factors in the form of cytokine receptor-like molecules that impair the inflammatory processes by intercepting signalling molecules [83] and it’s possible that these factors work more efficiently in blood than in an environment such as skin.

The broad range of specificities displayed by the ORFV-CBP suggests that this factor establishes a blockade to shield virus-infected cells from a wide range of immune cells [68,126,127]. The ability to bind CXC chemokines and lymphotactin may help limit the large influx of polymorphs that is associated with ORFV infection. In addition the ability of ORFV-CBP to bind CC chemokines and lymphotactin suggests that lymphocytes, B cells, NK cells and dendritic cells are of particular significance during infection. Other poxviruses also target the CXC and C classes of chemokine albeit with low affinity chemokine binding factors such as Crm-D and MT7 that are not structurally related to poxvirus CBP-II proteins [83]. No such homologues of these genes exist in ORFV and it would appear that evolutionary pressures have forced significant change to the ORFV-CBP gene to broaden its specificity to compensate for the lack of such factors.

Several studies have shown that ORFV produces a memory response [6,29]. This infers that ORFV has the ability to at least temporarily replicate in the immune host and suggests that it is able to subvert the reactivation of memory T cells during reinfection. During the course of the immune response, dendritic cells are recruited to the site of infection, capture antigen and migrate to the peripheral nodes where they present antigen to naïve T cells or memory T cells. ORFV has the potential to disrupt this process at multiple levels as the ORFV secreted factors IL-10, GIF, and CBP work in concert. ORFV-CBP could have a role in blocking the entry of dermal dendritic cells to the site of infection as well as their migration to peripheral lymph nodes since these processes are dependent on inflammatory chemokines and constitutive chemokines. Dendritic cells have been observed to accumulate at the site of ORFV infection [6] and it’s possible that this occurs as a result of ORFV-IL-10 blocking their maturation. This would also have the effect of blocking the upregulation of the CCR7 receptor that is required for migration of mature dendritic cells to peripheral lymph nodes in response to the constitutive chemokines [127]. In addition GM-CSF and TNF-α have been shown to be involved in the recruitment of dendritic cells to ovine dermis and in supporting the survival and proliferation of afferent lymph veiled dendritic cells [138]. Cytotoxic T cell migration from lymph nodes to the site of infection is also dependent on lymphotactin that ORFV-CBP binds with high affinity. Huang *et al.* [197] have shown that CD8+ T cell infiltration into tumors is enhanced by the expression of lymphotactin and it is noteworthy that CD8+ cells appear to become trapped under ORFV lesions [136] suggesting that the specificity of the ORFV-CBP for this chemokine may provide an explanation for this observation.

IFN-γ is associated with anti-viral immune responses and all poxviruses have evolved mechanisms to limit its action [83]. IFN-γ is produced by CD4+, CD8+ and has various effects on cells. Most poxviruses sequester IFN-γ by producing soluble IFN-γ -like receptor proteins. Although ORFV does not encode such a factor, it has the potential to suppress the production of IFN-γ since this cytokine is inhibited in NK cells, CD4+ Th1 cells and CD8+ cells by mammalian IL-10. Moreover IL-2 and IFN-γ have been implicated in protective immunity to ORFV reinfection [30,31,135,137] and both cytokines are targeted by ORFV factors GIF and IL-10 respectively. These observations strongly suggest that ORFV has evolved multiple mechanisms to disrupt the development of the adaptive responses. Moreover a delay in the mobilisation of the adaptive responses in the immune host by the viruses immune subversion mechanisms may explain its ability to reinfect its host after primary infection.

The accumulating evidence strongly suggests that ORFV factors manipulate keratinocytes either by inducing their proliferation or manipulating the cell into a stage of the cell cycle that best supports viral replication. Significantly ORFV has only been observed to replicate in actively dividing keratinocytes where cells are regenerating as a result of tissue damage. The differentiated keratinocytes that ORFV infects, in or near the stratum spinosum, are largely quiescent in undamaged tissue and the role of PACR could be to activate these cells into pseudo-S phase of the cell cycle as infection spreads away from the initial site of infection. In addition the VEGF may enhance viral growth by maintaining the regenerative response of damaged skin by directly promoting the growth of quiescent cells and increasing the supply of critical nutrients through the vasculature. It’s possible that the viral VEGF fulfils the same role as viral epidermal growth factor that is produced by other poxviruses that is associated with localised cellular proliferation. The increased vascular supply must also be important, as it would appear that the CBP binding specificities have evolved to avoid interaction with pro-angiogenic chemokines such as CXCL8 [198]. The ability of the viral VEGF to induce epidermal hyperplasia and dermal oedema may also contribute to walling off immune cells and scab formation that provides protection for the virus from environmental inactivation.

This brief summary of the known virulence genes of ORFV illustrates that it has evolved a package of factors that together conduct a co-ordinated inhibition of innate and adaptive immune responses while inducing a vascularised and proliferative cellular environment that supports viral replication. This package of ORFV factors appears to be particularly tuned to skin.

In the last 25 years, genetic and molecular research on ORFV has revealed some remarkable genes involved in replication, immune subversion and angiogenesis. Even more remarkable is that many of these genes do not appear to have any obvious evolutionary relationship to other poxvirus genes although there could be very distant relatives. In some cases virulence genes have been captured from the host during the evolution of the parapoxviruses. There still remain a number of ORFV genes with unknown functions. In time the role of these genes will be revealed and provide us with further evolutionary insight into this intriguing virus and its adaptation to skin.
